# Pretreatment with Sodium Phenylbutyrate Alleviates Cerebral Ischemia/Reperfusion Injury by Upregulating DJ-1 Protein

**DOI:** 10.3389/fneur.2017.00256

**Published:** 2017-06-09

**Authors:** Rui-Xin Yang, Jie Lei, Bo-Dong Wang, Da-Yun Feng, Lu Huang, Yu-Qian Li, Tao Li, Gang Zhu, Chen Li, Fang-Fang Lu, Tie-Jian Nie, Guo-Dong Gao, Li Gao

**Affiliations:** ^1^Department of Neurosurgery, Tangdu Hospital, The Fourth Military Medical University, Xi’an, China; ^2^Research Center of Traditional Chinese Medicine, Xijing Hospital, The Fourth Military Medical University, Xi’an, China

**Keywords:** DJ-1, ischemia/reperfusion injury, mitochondrial function, neuroprotection, sodium phenylbutyrate, reactive oxygen species

## Abstract

Oxidative stress and mitochondrial dysfunction play critical roles in ischemia/reperfusion (I/R) injury. DJ-1 is an endogenous antioxidant that attenuates oxidative stress and maintains mitochondrial function, likely acting as a protector of I/R injury. In the present study, we explored the protective effect of a possible DJ-1 agonist, sodium phenylbutyrate (SPB), against I/R injury by protecting mitochondrial dysfunction *via* the upregulation of DJ-1 protein. Pretreatment with SPB upregulated the DJ-1 protein level and rescued the I/R injury-induced DJ-1 decrease about 50% both *in vivo* and *in vitro*. SPB also improved cellular viability and mitochondrial function and alleviated neuronal apoptosis both in cell and animal models; these effects of SPB were abolished by DJ-1 knockdown with siRNA. Furthermore, SPB improved the survival rate about 20% and neurological functions, as well as reduced about 50% of the infarct volume and brain edema, of middle cerebral artery occlusion mice 23 h after reperfusion. Therefore, our findings demonstrate that preconditioning of SPB possesses a neuroprotective effect against cerebral I/R injury by protecting mitochondrial function dependent on the DJ-1 upregulation, suggesting that DJ-1 is a potential therapeutic target for clinical ischemic stroke.

## Introduction

Cerebral stroke is an important and tragic event that ranks as the second cause of death in the population. The number of incident strokes and stroke-related deaths has remained progressively increased in the past two decades (1). Cerebral stroke is defined as the sudden onset of loss of focal neurological function due to infarction or hemorrhage in the relevant part of the central nerve system, and ischemic stroke accounts for 70% of all stroke incidents (2). The best treatment for ischemic stroke is timely recanalization of the responsible artery followed by revascularization of the relevant area of the brain to salvage the peri-infarct neurons (3); however, ischemic/reperfusion (I/R) injury reduces the curative effect of this specific treatment. Therefore, to identify an efficient way to prevent I/R injury is essential for stroke patients to have a better outcome.

Cerebral I/R injury is a complex process that involves several mechanisms, including three major pathways in neurons: excitotoxicity, oxidative stress, and inflammation (4). Mitochondria play a key role in these pathways, both through ATP generation failure and as a key mediator in cell death pathways (5). In addition, I/R injury leads to mitochondrial dysfunction, which leads to oxidative stress and apoptosis (6). Thus, targeting mitochondria may be a potential therapy to reduce I/R injury.

DJ-1 is one of the causative genes associated with a familial form of Parkinson’s disease and has recently been proven to be a mitochondrial protector in I/R injury in the heart (7, 8). DJ-1 is critical for mitochondrial function, and the loss of DJ-1 causes mitochondrial fragmentation and dysfunction (9, 10). Hence, maintaining the expression and function of DJ-1 might be a promising way to protect mitochondrial function and further relieve I/R injury.

**Figure 1:**
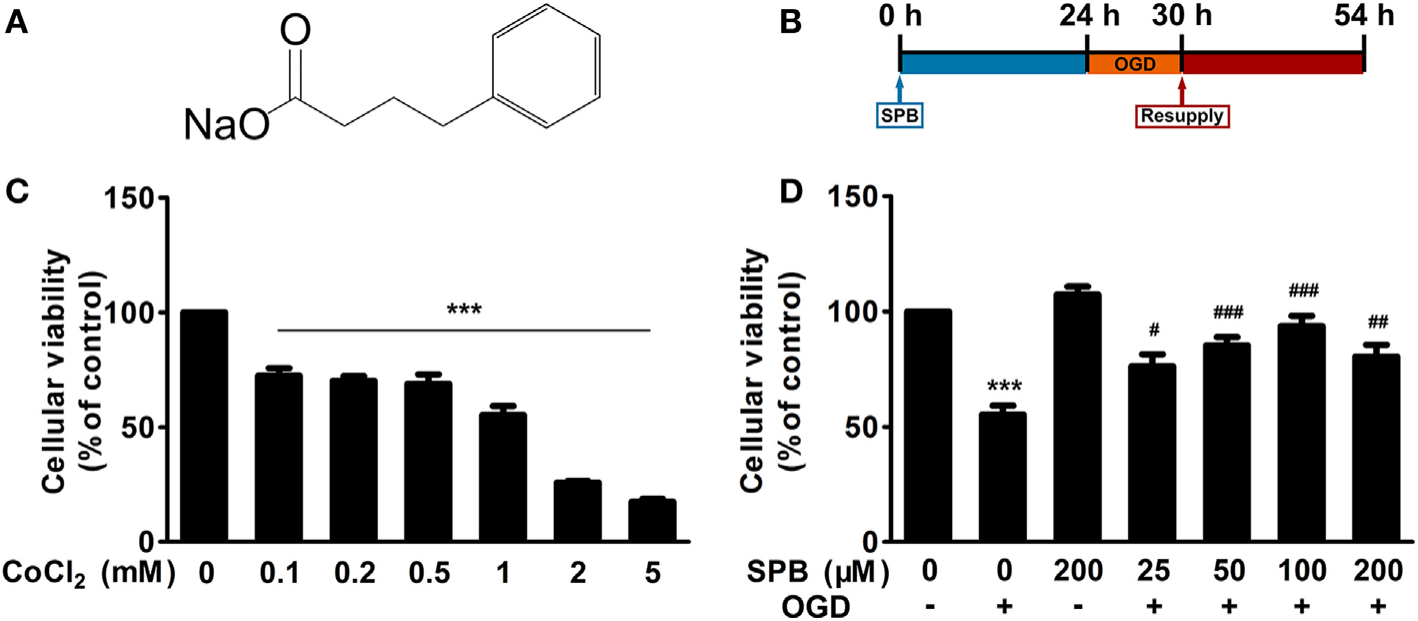
Sodium phenylbutyrate (SPB) improves cellular viability from ischemia/reperfusion (I/R) injury in a CoCl_2_-induced oxygen glucose deprivation (OGD) cell model in SH-SY5Y cells. **(A)** Chemical structure of SPB. **(B)** Experimental protocol of SPB treatment and cellular I/R model. SH-SY5Y cells were treated with SPB for 24 h and then were administered OGD by CoCl_2_ along with no-glucose culture medium for 6 h, followed by culturing in 10% FBS with DMEM for 24 h in a normal cell incubator to mimic reperfusion. This protocol was used in all cellular experiments unless otherwise indicated. **(C)** Effect of different concentrations of CoCl_2_ on the cellular viability of SH-SY5Y cells as assessed by the MTT assay. The procedure of this experiment was described above, except for no SPB treatment before OGD and different concentrations of CoCl_2_. ***p < 0.001, one-way analysis of variance (ANOVA), *n* = 4. **(D)** The protective effect of graded concentrations of SPB on CoCl_2_-induced I/R injury. CoCl_2_ (1 mM) was used to perform OGD. Cellular viability was measured by the MTT assy. ***p < 0.001, vs. the control group; ^#^*p* < 0.05, ^##^*p* < 0.01, ^###^*p* < 0.001, vs. OGD without the SPB group, one-way ANOVA, *n* = 4.

Sodium phenylbutyrate (SPB) is a small molecule (chemical structure shown in Figure 1A) that inhibits histone deacetylase activities and promotes the transcription of several genes, including that of DJ-1, in a Parkinson’s disease model (11). In this study, we investigated whether SPB exerts neuroprotection against I/R injury both in cell and animal models and explored the mechanisms underlying this protective effect.

## Materials and Methods

### Animals

All experimental protocols were designed according to the Guide for the Care and Use of Laboratory Animals (National Institutes of Health, USA, 2011) and were accepted by the Committee for Animal Care and Use of the Fourth Military Medical University (protocol no. SYXK 2012-001, Xi’an, Shaanxi, China). Male C57BL/6 mice, 4–8 weeks old with a body weight of 20–28 g, were purchased from the Experimental Animal Center of the Fourth Military Medical University (Xi’an, Shanxi, China). Animals were provided with commodious living conditions and standard food and water *ad libitum*. The housing environment was controlled at 21 ± 2°C, 40–60% humidity, and a 12-h dark/light cycle. Totally 90 mice were used in the experiments, and they were randomly divided into three groups: SHAM group, vehicle group, and SPB group (*n* = 30/group). The mice in the vehicle group and SPB group have been observed in survival tests (*n* = 30/group). Mice survived after middle cerebral artery occlusion (MCAO) were used for neurological deficits assessment (*n* = 24/29). After neurological function tests, some of the mice were used for lesion volume measurement (*n* = 11/group), and others were used for TdT-mediated dUTP nick end labeling (TUNEL) staining, immunofluorescent assay (*n* = 7/group), and other biochemical experiments (*n* = 6/group). Totally 10 animals died during the experiments and overall mortality was 11.1%, and all survival animals were sacrificed 7 days after MCAO. The *n* numbers were calculated according to previous descriptions (12, 13).

### Cell Culture and *In Vitro* I/R Model

The SH-SY5Y cell line was cultured in Dulbecco’s Modified Eagle’s medium (DMEM, Corning, NY, USA) with 10% (v/v) fetal bovine serum (FBS, Corning, NY, USA) at 37°C in 5% CO_2_/95% air. Most of the cellular experiments were administered when the cells reached 50–60% confluence.

For the cellular I/R model, a chemical oxygen glucose deprivation (OGD) model with CoCl_2_ was used in this experiment as described previously (14, 15). Cells were treated with CoCl_2_ and DMEM (glucose-free) to mimic ischemia for 6 h, and then, the culture medium was replaced with DMEM with 10% FBS for 24 h to mimic reperfusion. After 24 h of culture at 37°C, the cells were harvested for further experiments.

### Drug Treatment

Sodium phenylbutyrate was purchased from Sigma-Aldrich Company, USA. Totally 50 mice were equally and randomly divided into two groups: the SPB (120 mg/kg)-treated group and vehicle-treated group. SPB was dissolved in 0.9% saline and was injected intraperitoneally (i.p.) into mice in the treated groups mentioned above per day. The vehicle mice were treated with the same volume of natural saline. These treatments lasted for 7 days, and then, the mice were subjected to the MCAO model on the eighth day.

For the treatment of cells, SPB was dissolved in Dulbecco’s phosphate-buffered saline at the concentration of 100 mM and then was stored at −20°C until ready for use. Regarding cells in the SPB-treated group, SPB solution was added into the culture medium at an appropriate final concentration for 24 h, and then, it was discarded before OGD or transfection.

### Focal Cerebral I/R Mouse Model

The MCAO model was used in our experiment. The mice were fasted for 12 h before MCAO modeling, and then, they were anesthetized by a mixture of 70% N_2_O, 30% O_2_, and 2.0–3.0% isoflurane (RWD, GD, China) for induction or 1.0–1.5% isoflurane for maintenance (16, 17). A heating pad was used to keep the body temperature of the mice at 37–37.5°C during anesthesia induction, operation, and anesthesia recovery. Surgery was performed according to the procedure described previously (18). Briefly, after a midline skin incision on the neck, the common carotid artery was exposed. Next, a nylon monofilament (8-0) coated with silicone (Dongao Biosciences, SX, China) was inserted into the common carotid artery and was gently advanced along the internal carotid artery until the middle cerebral artery (MCA) had been occluded. A laser Doppler flowmeter was used to monitor the blood flow of MCA. After 1 h of occlusion without anesthesia, the filament was removed with mild anesthesia of the mice to establish reperfusion. The mice were kept on the warming pad to recover from the anesthesia and then were returned to the housing area. Mice in which the common carotid artery was exposed but not occluded were used as sham-operated controls.

Before subsequent experiments, animals were excluded according to the following criteria: (1) animals that died during anesthesia or surgery; (2) animals that died after surgery before further experiments; (3) animals in which the MCA blood flow was >20% of baseline after filament placement during the surgery; or (4) animals in which the MCA blood flow was <85% of baseline after filament removal.

### Assessment of Neurological Function, Infarct Volume, and Brain Edema

Neurological function, the infarct volume, and brain edema were assessed 24 h after MCAO surgery. Neurological deficits was assessed by the modified Bederson scoring system according to which was described by Hara et al. previously (16): 0, no observable neurological deficits (normal); 1, failure to extend right forepaw (mild); 2, circling to the contralateral side (moderate); 3, loss of walking or righting reflex (severe). The scoring was performed independently by two observers who were blinded to the groups. After scoring, the animals were deeply anesthetized, and the brains were quickly removed and then sectioned into five 2-mm slices using a mouse brain fixator and blades. The slices were incubated in 2% 2,3,5-triphenyltetrazolium chloride (TTC, Sigma-Aldrich, USA) for 30 min. Next, they were placed into 4% paraformaldehyde (w/v) and captured as digital images. ImageJ (ver. 1.43 h; National Institutes of Health, USA) was used to measure the infarct area and each hemisphere separately. Two investigators who were blinded to the groups measured the images independently. The infarct and edema volumes were calculated as previously described (19).

### Preparation of Paraffin Sections

Mice were deeply anesthetized by pentobarbital sodium 24 h after MCAO surgery and then were perfused with prechilled phosphate-buffered saline (PBS). Next, 4% paraformaldehyde (w/v) perfusion was administered for fixation until the color of the liver changed to light brown. The brains were then removed and dehydrated with graded concentration series of alcohol followed by paraffin embedding. The paraffin-embedded tissues were sectioned using a microtome (Leica, Germany) into 4-µm coronal slices. The slices that included the peri-infarct regions were selected and adhered to positron-conjugated slides. Before further experiments, the sections were stored at room temperature.

### TUNEL Staining

The TUNEL assay was performed using a cell death detection kit (Roche, Germany). After dewaxing and dehydrating the slides, 0.3% Triton X-100 (v/v) was used for permeabilization of the tissue samples on ice for 15 min. The TUNEL staining mixture was then added and incubated at 37°C for 60 min. After three washes with PBS, the sections were stained with DAPI and observed using a confocal microscope (C2 Si; Nikon, Japan). Regarding the cell samples, they were cultured on poly lysine-coated slides in 24-well plates. Next, 0.1% Triton X-100 (v/v) was used for permeabilization for 2 min on ice. The remaining procedures were performed as those for the tissue samples.

The TUNEL-positive cells were labeled with green fluorescence, and all of the cells were labeled by DAPI staining. The cellular apoptosis level was compared using the ratio of TUNEL-positive cells to all cells.

### Immunofluorescent Assay

After dewaxing and dehydrating, the sections of tissue samples were boiled with citrate buffer (pH 6.0) for antigen retrieval. Next, a mouse antibody against NeuN (1:100, Millipore, USA) and a rabbit antibody against DJ-1 (1:150, CST, USA) diluted with an antibody diluent, which contained 0.3% Triton X-100 (v/v) and 1% BSA (w/v), were incubated with the slides at room temperature for 12 h. Thereafter, the sections were incubated in a mixture of DyLight^®^ 488-conjugated goat anti-mouse IgG (1:200, ImmunoReagents, USA) and DyLight^®^ 594-conjugated goat anti-rabbit IgG (1:200, ImmunoReagents, USA) diluted in PBS. After DAPI staining, the slides were observed and captured using a confocal microscope (C2 Si; Nicon, Japan).

Regarding the cell samples, a rabbit antibody against DJ-1 (1:200, CST, USA) diluted in the antibody diluent was incubated with the slides with attached cells. The remaining procedures were performed as those for the tissue samples.

### Assessment of Cellular Viability

Cellular viability was detected by the methyl thiazolyl tetrazolium (MTT, Sigma-Aldrich, USA) assay. In short, SH-SY5Y cells were cultured in a 96-well plate and were treated as indicated. Next, MTT was added to each well and was incubated at 37°C for 2–4 h. The absorbance was read at 570 nm using a microplate reader (Bio-Rad, USA). The cellular viability was displayed as a percentage of the absorbance value (treated wells/control wells).

### Western Blot Analysis

For tissue samples, mice were deeply anesthetized and then were perfused by prechilled PBS. The brains were quickly removed, and the peri-infarct regions were isolated according to previous descriptions (20). Regarding the cell samples, after three washes with PBS, the cells were collected in a new 1.5-mL tube. The brain and cell samples were homogenized in lysis buffer with protease inhibitor. After the lysates were centrifuged, the supernatants were separated using SDS-PAGE, followed by transfer to polyvinylidene fluoride membranes. The membranes were blocked by 2% bovine serum albumin (Sigma-Aldrich, USA) for 1 h at room temperature and then were incubated at 4°C overnight with the antibodies against DJ-1 (1:1,000 dilution, CST, USA) and β-actin (1:3,000 dilution, Abcam, USA). The secondary antibodies conjugated with HRP were incubated with the membrane the next day. The bands were detected using an imaging system (Bio-Rad, USA) and were analyzed using ImageJ.

### DJ-1 siRNA Transfection

After treatment with or without SPB, the cells were transfected with DJ-1 siRNA (HSS117569, Invitrogen, USA) or control siRNA (12935200, Invitrogen, USA) using Lipofectamine 2000 (Invitrogen, USA) for 48 h according to the protocol provided by the manufacturer.

### Isolation of Mitochondria from Brain Tissue

The mitochondria isolation assay was performed using a tissue mitochondria isolation kit (Beyotime Biotechnology, JS, China). Fresh brain tissues containing the peri-infarct regions were quickly removed from deeply anesthetized mice. According to the manufacturer’s instructions, mitochondrial extraction buffer was added to the samples. Next, the mitochondria were isolated *via* gradient centrifugation. All of these procedures were required to be administered on ice to ensure the activity of the mitochondria.

### Reactive Oxygen Species (ROS) Detection

CM-H2DCFDA (Invitrogen, USA) was used to detect the ROS levels in both cells and isolated mitochondria from tissue. For each assigned cell group, the cells were incubated with 5 µM dye at 37°C for 30 min in a humidified incubator. Three washes with PBS were necessary to remove the excess dye. The cells at the same density that was quantified by cell counting were prepared in a microplate, and the intensity of fluorescence was measured using a multifunctional microplate reader (Bio-Rad, USA) with 485 nm excitation and 526 nm emission filters. As had been described before (21, 22), H2DCFDA (Invitrogen, USA) and a respiration buffer, including 5 mM pyruvate and 2.5 mM malate, was used during the incubation of isolated mitochondria. The amount of mitochondria was quantified by protein quantification. The measurement of the fluorescence levels were the same as that described above. Negative controls were set, in which cell samples or mitochondrial samples were omitted, to avoid interference from background ROS and residual dye.

### Mitochondrial Membrane Potential (MMP) Measurement

A MMP assay kit with JC-1 (Beyotime Biotechnology, JS, China) was used in this experiment. According to the manufacturer’s instructions, isolated mitochondria or cell samples were incubated with JC-1 staining-buffer, and then the fluorescence intensity was measured by a fluorescence spectrophotometer (490 nm excitation and 530 nm emission).

### Statistical Analysis

Data were analyzed by GraphPad Prism-5 (La Jolla, CA, USA). The survival data were analyzed using the log-rank (Mantel–Cox) test and Gehan–Breslow–Wilcoxon test. For the normally distributed data, Student’s *t*-test was used to assess differences between two groups. One-way analysis of variance (ANOVA) was used to compare differences among more than two groups, and Bonferroni *t*-test was used as a posttest in specific group comparisons. Mann–Whitney test was used to analyze non-normally distributed data. All values are shown as the mean ± SEM of at least three independent experiments. A *p* value < 0.05 was considered to have statistical significance.

## Results

### SPB Improves the Cellular Viability from I/R Injury in a CoCl_2_-Induced OGD Cell Model

To explore whether SPB can protect neurons from I/R injury, an OGD cell model induced by CoCl_2_ was performed in SH-SY5Y cells, a human neuroblastoma cell line, to mimic I/R injury *in vitro* as mentioned previously (15), and the protocol is displayed in Figure 1B.

We first treated cells with a range of CoCl_2_ concentrations for 6 h with simultaneous glucose deprivation, and then normal culture medium was resupplied to the cells for 24 h. Evaluated using the MTT assay, the cellular viability presented a dose-dependent reduction (Figure 1C). Cells that were exposed to 1 mM CoCl_2_ showed a reduction in cellular viability by approximately 50% (55.22 ± 3.94) compared with that in the control group. Therefore, the treatment in which 1 mM CoCl_2_ with glucose deprivation was administered for 6 h was used in the following experiments.

To determine the protective effect of different concentrations of SPB on cells, we next used graded concentrations of SPB-pretreated SH-SY5Y cells followed by CoCl_2_-induced OGD treatment as expounded above. A 200-µM SPB-treated group without OGD was set to observe whether SPB treatment influenced cellular viability independently. The data in Figure 1D showed that treatment with SPB alone slightly elevated cellular viability, but the difference was not statistically significant. However, the cellular viability was improved in all SPB-pretreated groups compared with that in the OGD group (Figure 1D). The protective effect was increased along with the concentration of SPB changing from 25 to 100 µM, but the effect was decreased slightly at a higher concentration of 200 µM. All of these results showed that SPB, especially at a concentration of 100 µM, improved cellular viability *in vitro*.

### SPB Dose-Dependently Increases the DJ-1 Protein Level and Rescues the DJ-1 Decrease Induced by OGD *In Vitro*

**Figure 2:**
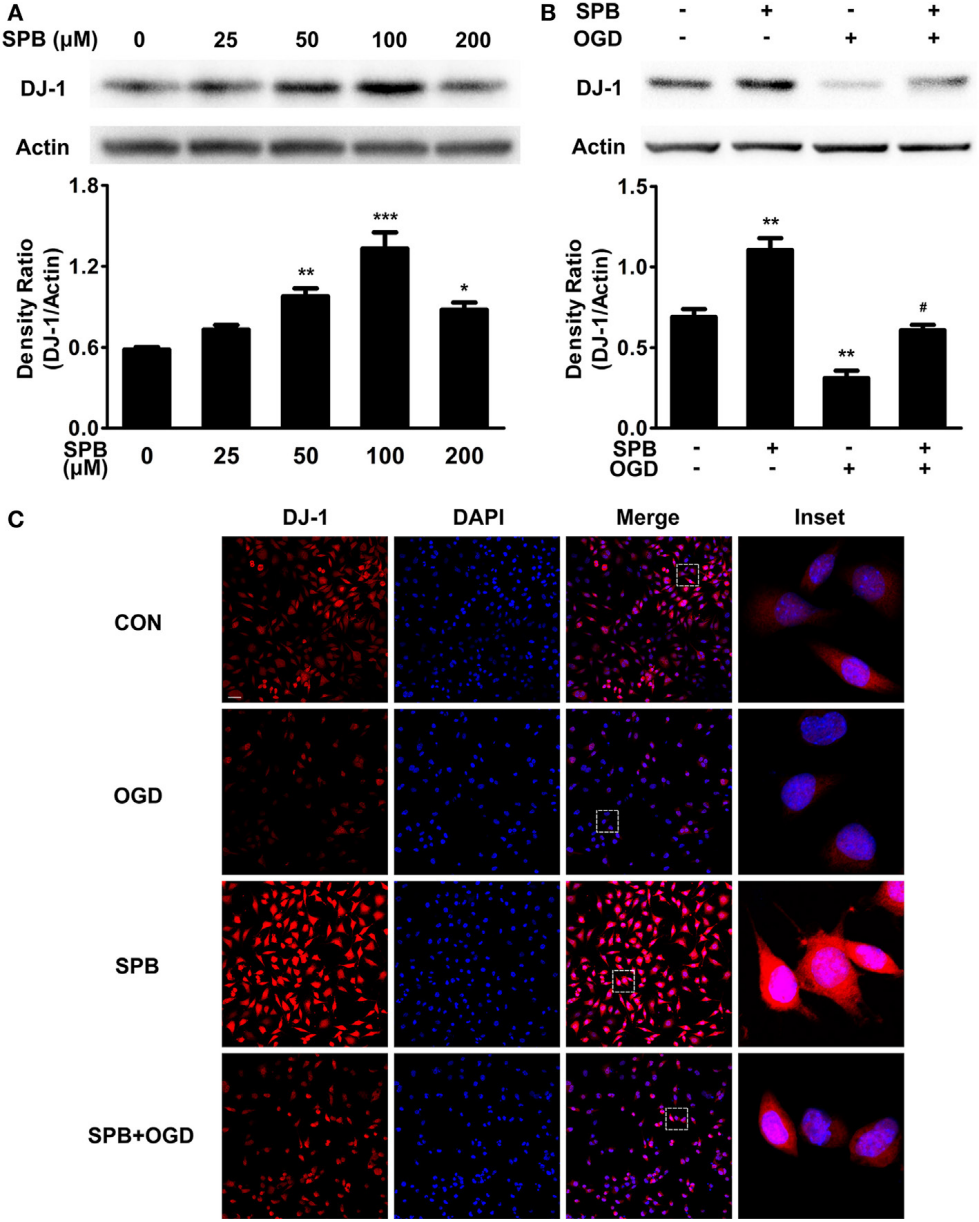
Sodium phenylbutyrate (SPB) dose-dependently increases the DJ-1 protein level and rescues the DJ-1 decrease induced by oxygen glucose deprivation (OGD) in SH-SY5Y cells. **(A)** Western blot analysis of the DJ-1 level in SH-SY5Y cells treated with different concentrations of SPB. Cells were treated with SPB for 24 h and were harvested for analysis. Quantitation data were shown as the ratio of the density; *p < 0.05, **p < 0.01, ***p < 0.001, one-way analysis of variance (ANOVA), *n* = 3. Western blot **(B)** and immunofluorescent analyses **(C)** of the DJ-1 level in SH-SY5Y cells treated with SPB and OGD. Quantitation data of the western blotting assay are shown below in **(B)** as the ratio of the density; **p < 0.01, vs. the control group; ^#^*p* < 0.05, vs. OGD without the SPB group, one-way ANOVA, *n* = 3. For immunocytochemistry **(C)**, the bar represents for 100 µm, and the insets represent the boxed areas (red: DJ-1; blue: DAPI).

To reveal the mechanism underlying SPB protection, we focused on DJ-1, an endogenous antioxidant protein, because it was reported that SPB upregulated DJ-1 expression in a Parkinson’s disease model (11), and DJ-1 showed a protective effect on I/R injury (8, 23). The levels of DJ-1 in each group treated with different concentrations of SPB were measured using western blot analysis. SPB upregulated the DJ-1 protein level in a dose-dependent manner. Particularly, obvious upregulation was observed at a concentration of 100 µM (Figure 2A), which was consistent with the former cellular viability assay. Therefore, the concentration of 100 µM SPB was used in subsequent cellular experiments.

To test whether I/R injury affected the DJ-1 level, we next used CoCl_2_ to induce the OGD model described above to mimic I/R injury in cells and found a severe reduction of the DJ-1 level (Figure 2B) that was partially rescued by SPB pretreatment. Immunocytochemistry was performed to confirm the results (Figure 2C). Fascinatingly, OGD treatment decreased the cytoplasmic DJ-1 level more obviously than the decline in the nucleus, and SPB alleviated the reduction in both the cytoplasm and nucleus. Our data revealed that SPB upregulated the DJ-1 protein level under basal conditions and rescued the DJ-1 decrease induced by I/R injury.

### SPB Ameliorates Mitochondrial Impairment and Cellular Apoptosis Induced by OGD *via* DJ-1 Signaling *In Vitro*

**Figure 3:**
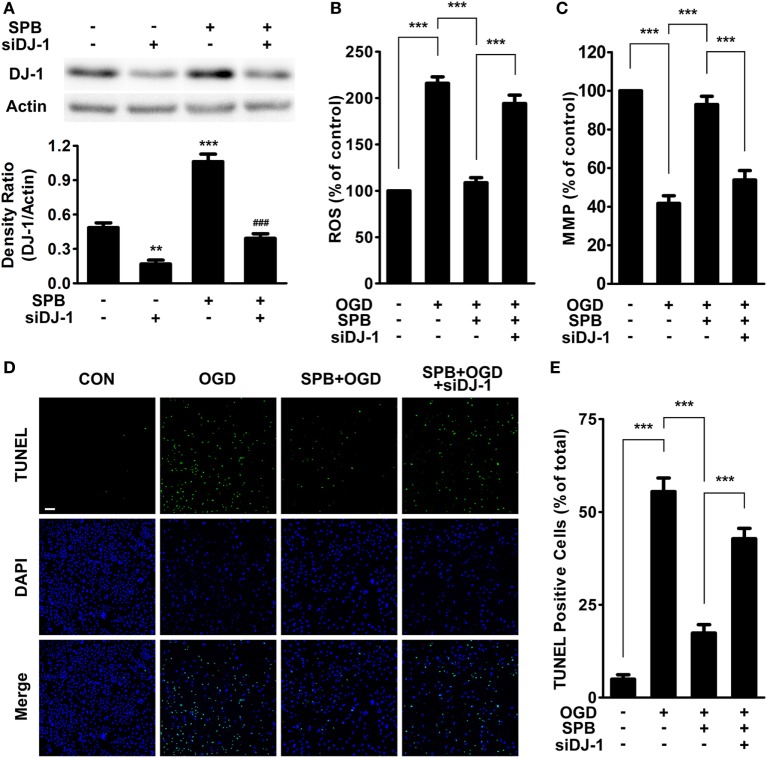
Sodium phenylbutyrate (SPB) ameliorates mitochondrial impairment and cellular apoptosis induced by oxygen glucose deprivation (OGD) *via* DJ-1 signaling in SH-SY5Y cells. **(A)** SH-SY5Y cells were treated with or without SPB and then were transfected with siRNA targeting human DJ-1 for 48 h, followed by western blot analysis to confirm the effective knockdown of the DJ-1 protein level. The graph below indicates the quantification of the DJ-1 level, **p < 0.01, ***p < 0.001 vs. the control group; ^###^*p* < 0.001 vs. the SPB-treated group; one-way analysis of variance (ANOVA), *n* = 3. Measurement of reactive oxygen species (ROS) production **(B)** and the mitochondrial membrane potential **(C)** in SH-SY5Y cells. Cells were pretreated with SPB for 24 h and were transfected with DJ-1 siRNA or control siRNA for 24 h, followed by OGD treatment as described in Figure 1B. ***p < 0.001, one-way ANOVA, *n* = 3. TdT-mediated dUTP nick end labeling (TUNEL) staining **(D)** and quantitative analysis **(E)**. The bar represents 200 µm (green: TUNEL; blue: DAPI). The treatments were the same as those described above. ***p < 0.001, one-way ANOVA, *n* = 4.

Because SPB upregulated the DJ-1 level and DJ-1 protected mitochondrial function as previously reported (24–27), we wondered whether SPB protected the cellular viability through maintaining mitochondrial function, and this protective effect was achieved *via* the upregulation of DJ-1 in our I/R injury model. Thus, we first set a DJ-1 knockdown model using DJ-1 siRNA transfection. After treatment with or without SPB for 24 h, SH-SY5Y cells were transfected with DJ-1 siRNA or control siRNA for another 48 h, and then, the cells were harvested for western blot analysis. The results showed that, following transfection with DJ-1 siRNA, the DJ-1 protein level was reduced by over 60% both in SPB-treated or untreated cells, compared with that in the control siRNA group (Figure 3A).

Next, we further detected the levels of ROS and MMP in cells to assess mitochondrial function. The results showed that the OGD model significantly increased the ROS level and decreased MMP (Figures 3B,C). However, after 24-h pretreatment with SPB, the cells showed milder mitochondrial impairment; less ROS production and a higher MMP level were detected under CoCl_2_-induced I/R injury. By contrast, reducing the DJ-1 level counteracted the protection of SPB against both MMP decrease and ROS production. This result suggests that SPB protected mitochondrial function through maintaining the DJ-1 protein level.

To determine whether SPB protected cell survival from I/R injury through the DJ-1-mitochondria pathway, we next used TUNEL staining to detect cellular apoptosis in a CoCl_2_-induced OGD model. Consistent with previous experiments, the pretreatment of SPB prominently reduced cell apoptosis compared with the OGD-alone-treated group (Figures 3D,E). Additionally, the downregulation of DJ-1 by siRNA crippled the protective effect of SPB against OGD-induced cell death.

As described above, SPB protected cellular viability and prevented cell apoptosis and mitochondrial dysfunction in the I/R injury model. However, all of these effects were impaired when DJ-1 was knocked down in SH-SY5Y cells. Thus, our results suggested that SPB protects cells from OGD injury *via* the DJ-1-mitochondria pathway *in vitro*.

### SPB Reverses the DJ-1 Decrease and Mitochondrial Dysfunction Induced by MCAO in Mice

**Figure 4:**
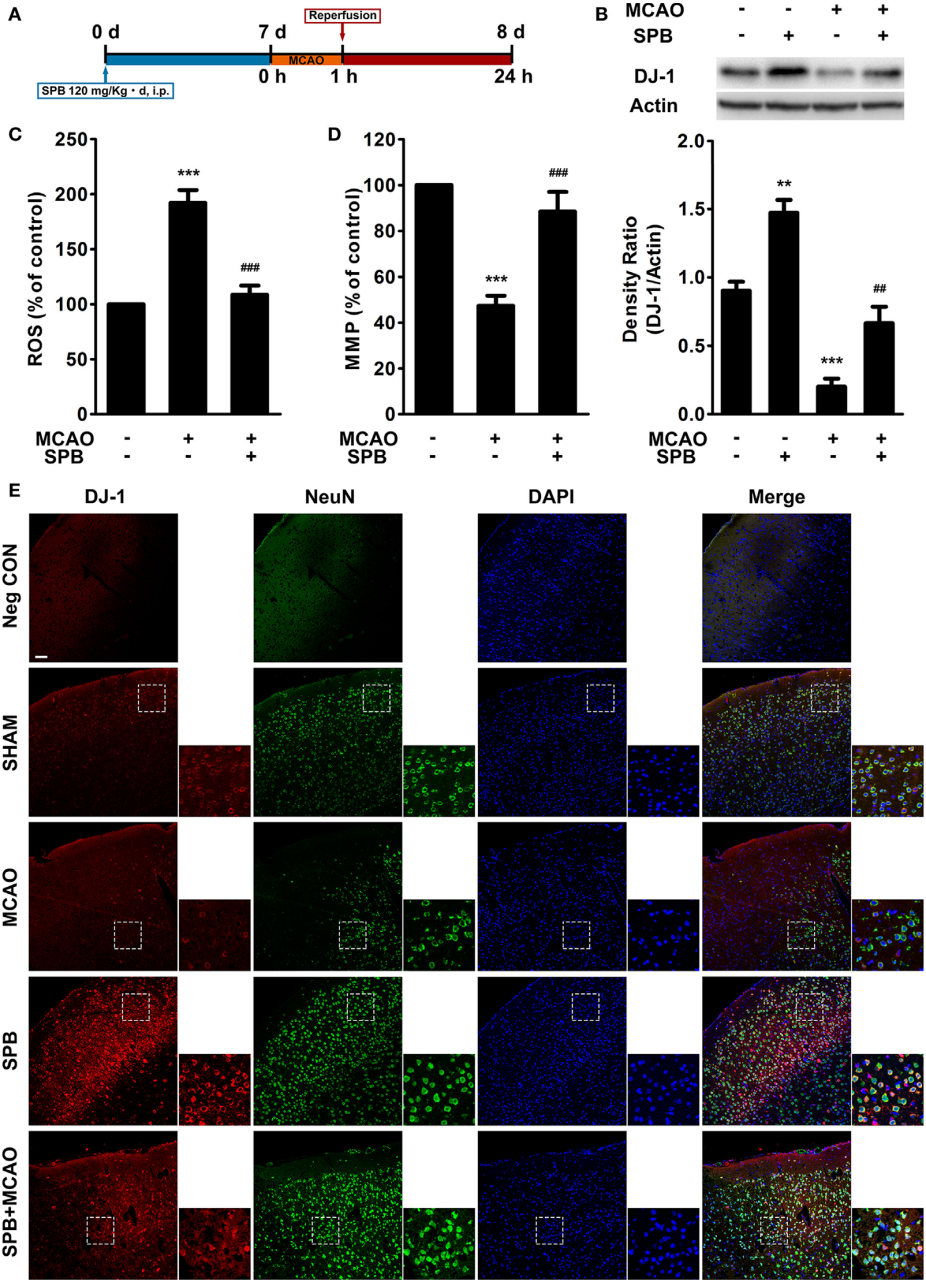
Sodium phenylbutyrate (SPB) reverses the DJ-1 decrease and mitochondrial dysfunction induced by ischemia/reperfusion (I/R) injury in mice. **(A)** Experimental protocol of SPB treatment and the I/R model in mice. SPB was injected intraperitoneally at 120 mg/kg per day for 7 days. Middle cerebral artery occlusion (MCAO) was administered for 1 h on the eighth day as described in the methods, and then, reperfusion was established for 23 h. All of the subsequent animal experiments were performed 23 h after reperfusion unless otherwise stated. **(B)** Western blotting of DJ-1 in the mouse brain and quantitative analysis. The samples were from the peri-infarct region of MCAO mice or relative regions of sham surgery mice. **p < 0.01, ***p < 0.001, vs. the control group; ^##^*p* < 0.01, vs. MCAO without SPB treatment group, one-way analysis of variance (ANOVA), *n* = 6. Measurement of reactive oxygen species (ROS) production **(C)** and the mitochondrial membrane potential **(D)** in the peri-infarct region or relative regions of mice. ***p < 0.001, vs. the control group; ^###^*p* < 0.001, vs. MCAO without SPB treatment group, one-way ANOVA, *n* = 6. **(E)** Immunofluorescence analysis of DJ-1 and NeuN colocalization. The coronal sections were serially sectioned from the plane on which hippocampus begins to appear, and the cortex region were observed and displayed in the figure. The smaller pictures show the details in the boxed areas (red: DJ-1; green: NeuN; blue: DAPI; bar for 200 µm).

To ascertain the protective role of SPB against I/R injury and its potential for clinical treatment, we used the MCAO mouse model, which is commonly used in I/R injury studies, to perform further studies; the protocol is shown in Figure 4A.

We first detected the DJ-1 level in mouse brains from the SHAM or MCAO groups with or without SPB treatment (120 mg/kg per day, for 7 days). The samples of MCAO mice were obtained from the peri-infarct regions, and the control samples were obtained from similar regions from sham surgery mice. Western blot analysis showed that SPB remarkably upregulated the DJ-1 protein level in mice with sham surgery (Figure 4B), a finding that was consistent with cellular experiments showing that SPB elevated the DJ-1 level under basal conditions. The decrease in the DJ-1 level induced by I/R injury was also observed in peri-infarct neurons in mice, and SPB rescued this effect as previously demonstrated in cell models (Figure 4B).

We next measured ROS production and the MMP of cells from peri-infarct regions to estimate mitochondrial impairment by MCAO. ROS production was decreased, and the MMP level was increased, reflecting a protective effect of SPB on mitochondria *in vivo* (Figures 4C,D).

We further observed whether SPB elevated the DJ-1 levels, especially in neurons. Using the immunofluorescent assay, we found more colocalization of NeuN, a neuronal marker, with DJ-1 in SPB-treated mice in the presence or absence of MCAO (Figure 4E). Intriguingly, since dead neurons could not be stained by NeuN, we found more NeuN-positive neurons in SPB-pretreated mice near the infarct area after MCAO compared with MCAO mice without SPB treatment. Moreover, in SPB-pretreated mice, the disturbed arrangements, but not death events, of neurons indicated that the neurons demonstrated I/R injury induced by MCAO but not as severe as what occurred in the mice without SPB treatment (Figure 4E). All these data showed that SPB had its effect, especially in neurons, in increasing DJ-1 expression, improving mitochondrial function, and possibly promoting neuronal survival in mice.

### SPB Attenuates Neuronal Apoptosis in Peri-Infarct Regions, Cerebral Infarction, and Brain Edema after MCAO

**Figure 5:**
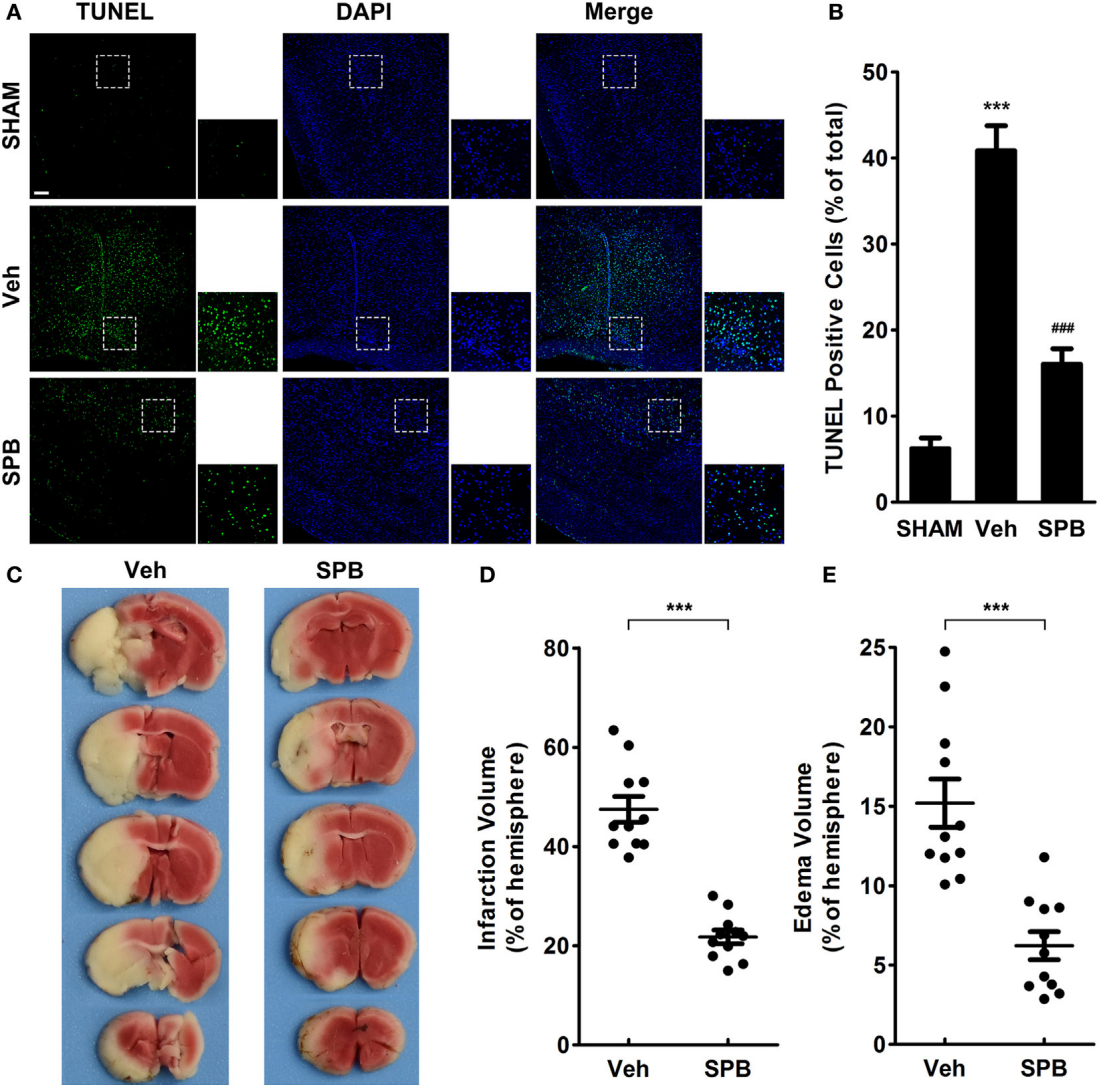
Sodium phenylbutyrate (SPB) attenuates neuronal apoptosis in peri-infarct regions, cerebral infarction, and brain edema after ischemia/reperfusion injury in mice. TdT-mediated dUTP nick end labeling (TUNEL) staining **(A)** and quantitative analysis **(B)** of coronal slices (4-µm-thick) of peri-infarct region. The bar represents 200 µm (green: TUNEL; blue: DAPI). The data were displayed as percentages of total cells, ***p < 0.001, vs. the SHAM group; ^###^*p* < 0.001, vs. the vehicle group, one-way analysis of variance, *n* = 6. **(C)** 2,3,5-Triphenyltetrazolium chloride (TTC) staining of 2-mm-thick coronal sections. Veh represents the vehicle group. Quantitative analysis of the infarct volume **(D)** and edema volume **(E)** in each section. The data were displayed as hemispheric percentages, ***p < 0.001, Student’s *t*-test, *n* = 11.

To confirm the phenomenon we observed in the NeuN staining assay that SPB may rescue neurons in peri-infarct regions from death after MCAO, we next used TUNEL staining to detect cellular apoptosis in the peri-infarct region. The results indicated that, as expected, SPB protected peri-infarct cells from I/R injury-induced cell death (Figures 5A,B). Additionally, in the TTC staining assay, which was performed to measure the infarct volume and brain edema after MCAO, we found that SPB pretreatment indeed decreased the infarct volume and relieved brain swelling (Figures 5C–E).

### SPB Promotes Mouse Survival and Alleviates the Neurological Deficit after MCAO

**Figure 6:**
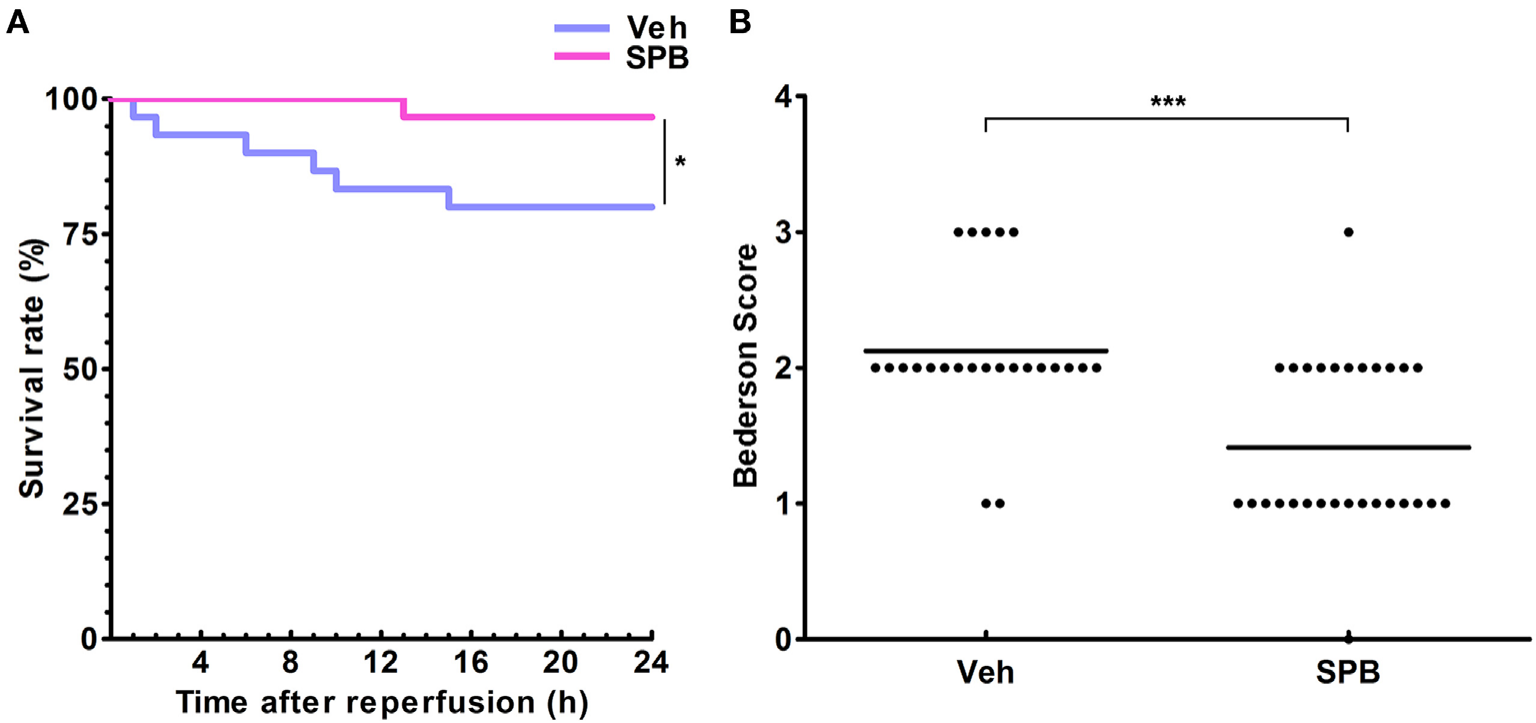
Sodium phenylbutyrate (SPB) promotes mouse survival and alleviates neurological deficits after ischemia/reperfusion injury in mice. **(A)** The survival curve of middle cerebral artery occlusion (MCAO) mice with or without SPB treatment. Mice were observed every hour after MCAO operation, and each death event was recorded immediately. *p < 0.05, log-rank (Mantel–Cox) test and Gehan–Breslow–Wilcoxon test, *n* = 30. **(B)** Neurological scores were assessed using the modified Bederson scoring system. ***p < 0.001, Mann–Whitney test, *n* = 24/29.

Finally, we observed the survival rate and neurological functions of mice. After MCAO and reperfusion, the mice were observed every hour to assess the occurrence of death events. The survival curve showed that SPB pretreatment, at 120 mg/kg per day, improved the survival rate 24 h after operation and delayed the first death event of MCAO mice compared with that in the vehicle group (Figure 6A). The neurological function assessed by the Garcia scoring system also showed that mice pretreated with SPB obtained better scores (Figure 6B). These data indicate that SPB pretreatment improves the survival rate and reduces the neurological deficits of mice after MCAO.

## Discussion

In this study, we have demonstrated the protective effect of SPB in both cell and animal models against I/R injury. In cell models, we have found that SPB upregulates the DJ-1 protein level in SH-SY5Y cells. The increased DJ-1 protein level leads to a higher resistance of the cells to OGD-mimicked I/R injury and a milder mitochondrial dysfunction induced by OGD. However, blocking the activation of DJ-1 by siRNA transfection reverses SPB protection. In mouse models, a higher DJ-1 protein level was found in the brain of SPB-treated mice. The pretreatment of SPB reduces the death rate and neurological deficits in MCAO mice because the infarct volume decreases and brain edema was alleviated *via* SPB treatment. All of these protective effects in SPB-treated mice may, at least partially, be due to the upregulation of DJ-1, and the activation of DJ-1 protects mitochondrial function, as proven in the cell models.

Sodium phenylbutyrate is a histone deacetylase inhibitor (HDACi) and can increase acetylation levels of histones H3 and H4, thereby promoting transcriptional activation (11, 28, 29). SPB can activate the expression of many genes including survival motor neuron 1, components of ubiquitin-proteosomal pathways, and adrenoleukodystrophy-related gene (29–32). With a low molecular weight, SPB is well distributed in the central nervous system (33), indicating that SPB has the medicinal basis used in nervous system disorders. In fact, a group has reported that SPB protects the brain against ischemia injury in mice (19). In this study, researchers have used a hypoxia-ischemia animal model using the right carotid artery ligated and 30 min of hypoxia at 6% O_2_. The group is convinced that the suppression of CHOP and inhibition of endoplasmic reticulum (ER) stress are the main protective pathways induced by SPB. Subsequently, another group reported that SPB ameliorated focal cerebral I/R injury associated with comorbid type 2 diabetes by reducing ER stress and DNA fragmentation (34). Considering their findings, the protective effect of SPB against cerebral I/R injury might be multifactorial and complicated, and more studies are needed to recover the central pathway underlying this effect. However, in these two studies, SPB were posttreated to demonstrate the therapeutic effect of SPB, and our study were performed using preconditioning of SPB to clarify its preventive effect against I/R injury. Furthermore, we consider that the mitochondria, as the energy factory of cells, play a crucial role in the first phase of I/R injury and might be more important in the pathways involved in the SPB protective effect.

The bioenergetics failure of cells occurs immediately after the blood flow is interdicted. Without ATP generation, calcium homeostasis is broken, causing the mitochondria to be more susceptible to various reflow-induced pathogenic events (6). Once reperfusion is established, oxidative stress, which indicates increased free radical formation and mitochondria membrane permeability, leads to more severe mitochondrial impairment (35), and cellular apoptosis ensues, similar to what we have observed in our experiments. Additionally, mitochondrial dysfunction accompanies the production of ROS (36). Excess ROS production has high toxicity to key cellular macromolecules, including DNA, proteins, and lipids, and ROS cause damage to mitochondria and may eventually lead to cell death (37). Therefore, preventive and therapeutic methods targeting mitochondria may be more efficient in stroke treatment. In this study, we have displayed that pretreatment of SPB ameliorates mitochondria impairment *via* reducing ROS production and elevating MMP, further decreasing the apoptosis of peri-infarct neurons. This neuroprotective effect indicates the possibility that pre-use of SPB may improve the patients’ neurological function after thrombolysis by preserving more peri-infarct neurons from I/R injury.

It has been reported that SPB, acting as a HDACi, can activate DJ-1 gene expression in a Parkinson’s disease model (11). DJ-1 gene, first discovered as an oncogene (38), was further identified as a causative gene for familial Parkinson’s disease PARK7 with recessive inheritance (39). DJ-1 is ubiquitously expressed in almost all tissues and has multiple functions such as transcriptional regulation, antioxidative stress reaction, and mitochondrial regulation (7). Recently, the protective effect of DJ-1 in cardiovascular diseases has been uncovered through a DJ-1-mitochondria pathway (8, 26). The mechanisms by which DJ-1 protects mitochondrial function have not been clearly exposed. The upregulation of nuclear factor erythroid-2-related factor 2 (Nrf2) transcriptional activity and direct translocation into the mitochondria are two pathways that may be involved in DJ-1 maintaining mitochondrial function (7). By sequestering Keap1, an Nrf2 binding protein in the cytosol promoting Nrf2 degradation (40), DJ-1 promotes Nrf2 binding to antioxidant response elements by which Nrf2 can regulate the expression of several endogenous antioxidative enzymes and reduce ROS production to protect mitochondria and can also respond to oxidative stress. Additionally, DJ-1 protects mitochondria by directly maintaining mitochondrial complex I activity (41) and translocating into mitochondria as an endogenous antioxidant (42). This evidence confirms the therapeutic potential of DJ-1 as a target in the clinical treatment of stroke because mitochondrial impairment plays an important role in I/R injury. Our study found that in both cell and animal I/R injury models, the DJ-1 level is decreased, followed by mitochondrial dysfunction and neuronal apoptosis. Thus, DJ-1 is essential for neurons to survive during I/R injury, and this standpoint is further confirmed in subsequent experiments in which SPB upregulates the DJ-1 level and improves neuron survival in I/R injury models.

Another interesting phenomenon is that in cell models, cytoplasmic DJ-1 is decreased more notably than nuclear DJ-1. We believe that this difference is due to the multiple functions of DJ-1. In the nucleus, DJ-1 usually acts as a transcription modulator (43, 44); however, in the cytoplasm, DJ-1 more easily quenches the activity of ROS and protects mitochondrial function (7). Thus, the reduction of DJ-1 in the cytoplasm may have a more direct influence on mitochondrial function. The mechanism by which I/R injury impairs the DJ-1 level is not clear. Does the expression of DJ-1 decrease or does the elimination of DJ-1 increase? We prefer the latter situation. DJ-1 may be oxidized during I/R injury-induced oxidative stress, and then, oxidized DJ-1 proteins are degraded through proteasome-mediated degradation and autophagy (45, 46). Therefore, upregulating DJ-1 both before and during I/R injury is supposed to be preventive and therapeutic methods for stroke because more DJ-1 can be preserved for other essential functions based on the former hypothesis. In this study, we have presented an effective way to upregulate DJ-1 levels. In both cell and animal models, SPB upregulates DJ-1 under normal and stress conditions. Additionally, in DJ-1 knockdown cells, SPB loses its protection against I/R injury, confirming that the neuroprotective effect of SPB occurs in a DJ-1-dependent manner. Preconditioning of SPB elevates DJ-1 level, which may give cells a higher resistance against I/R injury. This neuroprotection of SPB indicates its potential preventive usage in the group with high stroke risks. However, there are also some limitations of this kind of usage of SPB. In the existing treatment of SPB for children with urea cycle disorders, the longest using time of SPB is approximately 2 years, and there are no adverse effects observed in those children (47). But for the group with high stroke risks, it may take a much longer time to take SPB persistently, and the risks of long-term use are still unknown. We have to weigh the benefits of SPB against the long-term use risks before clinical usage and clarify the underlying mechanisms of the neuroprotective effects of SPB. Although there is a long way to go, we are still convinced that SPB owns a promising prospect in clinical use for stroke patients and the people at high risk of it. Actually, it has been reported that SPB can alleviate focal cerebral I/R injury in rats MCAO models associated with comorbid type 2 diabetes, which shows a great promise of SPB using as a preventive treatment for patients with stroke risk factors.

## Conclusion

In this study, we have demonstrated that pretreatment of SPB protects against I/R injury both *in vivo* and *in vitro*. Through upregulating DJ-1 protein, SPB maintains mitochondrial function and preserves neuron survival. Targeting DJ-1 and mitochondria may be a promising way to ameliorate I/R injury. Finally, we present the preventive potential of SPB in clinical usage in patients with high stroke risks, which may promise them a better neurological function and prognosis.

## Ethics Statement

All experimental protocols were designed according to the Guide for the Care and Use of Laboratory Animals (National Institutes of Health, USA, 2011) and were accepted by the Committee for Animal Care and Use of the Fourth Military Medical University (protocol no. SYXK 2012-001, Xi’an, Shaanxi, China).

## Author Contributions

YR-X, LJ, GG-D, and GL designed the study. YR-X, WB-D, and LC were responsible for MCAO model operating, TTC staining, and neurological scoring. LJ, LT, and LF-F performed cell culturing, the MTT assay, and western blotting. YR-X and LY-Q performed ROS and MMP detection. YR-X and NT-J performed TUNEL staining. LJ, HL, and ZG performed immunofluorescent assay and image analysis. YR-X, LJ, WB-D, FD-Y, and GL analyzed the data and discussed the results. YR-X, LJ, FD-Y, GG-D, and GL wrote the manuscript.

## Conflict of Interest Statement

The authors declare that the research was conducted in the absence of any commercial or financial relationships that could be construed as a potential conflict of interest.
