# Supplementary figures and images for: Pretreatment with Sodium Phenylbutyrate Alleviates Cerebral Ischemia/Reperfusion Injury by Upregulating DJ-1 Protein
Source: Front Neurol. 2017 Jun 9;8:256. doi: 10.3389/fneur.2017.00256 (PMC5465296; doi:10.3389/fneur.2017.00256)

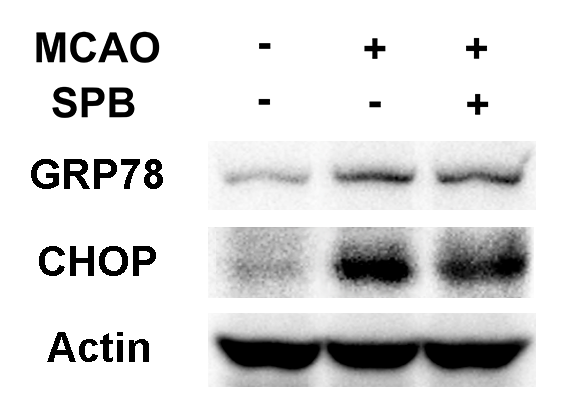

Supplement: Supplementary file 1 [file Image_1.TIF]

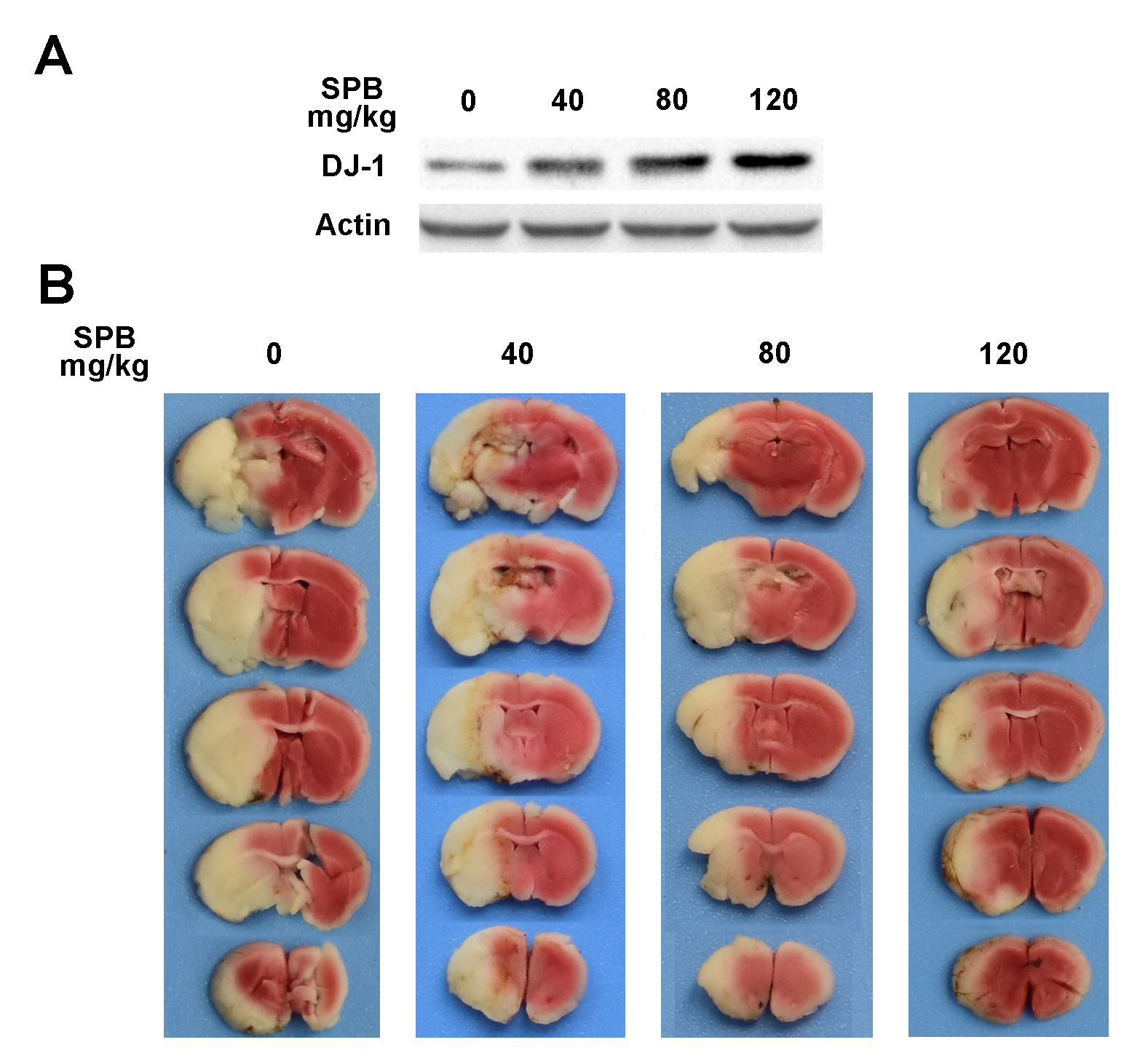

Supplement: Supplementary file 2 [file Image_2.TIF]
